# Emergency Department Pneumonia Patients Who do not Meet the Six-Hour Criteria for Antibiotic Administration: Do They Have a Different Clinical Presentation?

**DOI:** 10.4021/jocmr1092w

**Published:** 2012-09-12

**Authors:** Susan H. Watts, E. David Bryan

**Affiliations:** a Texas Tech University Health Sciences Center, Paul L Foster School of Medicine, Department of Emergency Medicine, USA

**Keywords:** Community acquired pneumonia, CAP, Joint Commission

## Abstract

**Background:**

The Joint Commission (JC) has set a quality of care standard for emergency department (ED) patients diagnosed with community acquired pneumonia (CAP) that states that they are to receive antibiotics within six hours of presentation to the ED. Hospitals have been able to demonstrate that the majority of patients meet these criteria, yet there are still many who do not. Previously published studies have reported that there are several issues that contribute to prolonged times to antibiotic administration including ED crowding and atypical clinical presentations. This study was undertaken to identify factors existing early in the patient encounter that may be associated with failure to meet the Joint Commission’s six-hour standard for antibiotic administration.

**Methods:**

This was an IRB-approved, retrospective observational study covering 36 months in an academic emergency department. All adults with an admission diagnosis of CAP were eligible but were excluded if their discharge diagnosis was not CAP, if hospitalized within the previous 14 days, or if HIV positive. Univariate analysis and multiple logistic regression with stepwise variable selection were performed comparing patients who met and did not meet JC standards. The analysis included demographics (age, sex), chief complaint at triage and to doctor (fever, dyspnea, cough, chest pain, weakness/fatigue, abdominal pain), presence of altered mental status, triage vital signs, co-morbidities, day of week and time of day of presentation.

**Results:**

A total of 736 cases were eligible; 199 cases met exclusion criteria; 43 charts were unavailable; 494 were included in the study group (363 with complete antibiotic time records; 131 were incomplete). From the univariate analysis, respiratory rate (RR) and oxygen saturation were the only factors that met Bonferroni criteria for statistical significance when comparing those who met and did not meet the JC six-hour criteria (RR 25 ± 9 vs 22 ± 6 breaths/minute, respectively, P = 0.002; oxygen saturation 87 ± 10% vs 92 ± 5%, respectively, P < 0.001). Multiple logistic regression identified triage pulse rate, oxygen saturation, presence of altered mental status, hour of day, and day of week as variables associated with time to antibiotic administration. Chances for meeting the standard were increased by 10% for each 5-beat increase in pulse rate or 1% decrease in oxygen saturation. If the person exhibited altered mental status, they were > 3.5 times more likely to meet the 6-hour criteria. If they presented to ED between 3 PM and 10 PM chances of meeting criteria were reduced by about 65%. If they presented on a Thursday, chances improved 2.8 times.

**Conclusions:**

Compared to patients who did meet Joint Commission criteria, those who did not receive antibiotics within 6 hours were likely to have triage pulse rates and O_2_ saturation levels closer to normal, thus contributing to diagnostic uncertainty. They were also likely to present to the ED at the most crowded time of day. Likelihood to meet JC criteria was improved if O_2_ saturation was below normal, pulse rate was elevated, if they exhibited mental confusion, or if they presented to the ED very early or very late in the day, or on a lower census day.

## Introduction

The length of time that elapses before patients diagnosed with community acquired pneumonia (CAP) receive antibiotics in the Emergency Department (ED) is thought to have an impact on patient outcome [1, 2]. Consequently, the Infectious Disease Society of America and the American Thoracic Society set time guidelines for the administration of the first dose of antibiotics to patients diagnosed with CAP in the ED [3]. Although there is little evidence to support a specific time recommendation for antibiotic administration, the Joint Commission on Accreditation of Healthcare Organizations (Joint Commission or JC) has instituted quality of care standards (namely core measures) for hospitals that evaluate whether pneumonia patients receive their first dose of antibiotics within six hours of hospital arrival (PN-5c) [3, 4]. This has been modified from an earlier standard (PN-5b) for which the time limit had been set at four hours.

Joint Commission criteria are met for the majority of ED patients diagnosed with CAP, but the treatment of many patients still fails to meet the six-hour standard. Previously published studies have reported that prolonged time to first dose of antibiotics is associated with many issues including atypical clinical presentations [5] and ED crowding [6, 7]. In our own ED, we have observed that the sickest patients appear to be treated in the shortest length of time, namely their clinical presentation is definitive and treatment can be initiated quickly. We hypothesized that those who have prolonged times for antibiotic administration were more likely to have clinical presentations that are more ambiguous, and therefore treatment is delayed.

The objective of this study was to identify factors existing early in the patient encounter that may be associated with the failure to meet the Joint Commission’s six-hour standard for antibiotic administration.

## Methods

### Study design, setting, and population

This retrospective observational study covering a 36-month period (2002 - 2004) was approved by the El Paso IRB of the Texas Tech University Health Sciences Center with waiver of consent. It was conducted in an academic emergency department with about 60,000 patient visits per year.

### Study protocol

All adult patients (≥18 years old) with a hospital admission diagnosis of CAP were eligible for inclusion. A list of potentially eligible patients was generated using the ED information system (Logicare Corporation, Eau Claire, WI) employing search terms for all pneumonia types including bacterial, viral, aspiration, and anaerobic pneumonias. This allowed for the inclusion of CAP patients who might have been miscoded due to clerical errors. One of the investigators (SHW) performed the chart reviews and recorded the information on an electronic data abstraction form (Microsoft Access, Microsoft Corporation, Redmond WA). Patient cases were excluded if the final hospital discharge diagnosis was not CAP, if they had been hospitalized within the 14 days prior to this admission, or if they were human immunodeficiency virus (HIV) positive. These criteria reduced the probability of including patients with hospital-acquired or atypical pneumonias and are consistent with previous CAP studies [8, 9].

Data abstracted from the charts included elements necessary to calculate Pneumonia Severity Index (PSI) [10],including demographics (age, sex, nursing home resident); co-morbidities (history of neoplasia, liver disease, congestive heart failure, cerebrovascular disease, or renal disease); triage vital signs (respiratory rate, systolic blood pressure, temperature, pulse, oxygen saturation), presence of mental confusion; worst vital signs (respiratory rate, systolic blood pressure, temperature, pulse, oxygen saturation); and results of some laboratory and imaging studies (arterial pH less than 7.35, PO^2^ less than 60 mmHg, sodium if less than 130 mEq/L, BUN if greater than 30 mg/dL, glucose if greater than 250 mg/dL, hematocrit if less than 30%, and presence of pleural effusion). In addition we recorded the patient’s chief complaint at triage and to the doctor (fever, dyspnea, cough, chest pain, weakness/fatigue, abdominal pain), times of blood culture orders and draws, times of antibiotic orders and administration, hospital unit admitted to, ED triage date and time, hospital discharge date and time, and final diagnosis. Time to antibiotic administration was calculated as the time between the electronic triage time stamp and time of antibiotic administration recorded in the chart. Presence of mental confusion was considered positive if the patient’s chief complaint or history of present illness included references to altered mental status (AMS), somnolence, or behavior changes. History of dementia or Alzheimer’s disease was not considered positive for mental confusion unless there was a change from their usual condition.

### Measurements

We limited our statistical analysis to data that would be available early in the patient encounter, namely before the results of lab and imaging studies would be available for most patients. This decision was based on the assumption that in many EDs there may be a significant delay between triage and physician examination and a further delay when awaiting laboratory and imaging results [11, 12]. Once the decision to give antibiotics has been made, orders must be written and transmitted and the drugs must be obtained and administered. Therefore, we reasoned that if the six-hour criteria are to be met, the decision to give antibiotics is often made soon after the initial patient encounter.

### Data analysis

Univariate statistical analysis was performed for each of the risk factors described below using T-tests for continuous variables and Fisher’s exact test for categorical variables (Stata, v.10.1; College Station, TX). Due to the large number of variables (n = 28), Bonferroni criteria were applied, making statistical significance P < or = 0.002 (namely 0.05/28). Multiple logistic regression was performed using stepwise variable selection to compare characteristics of those who met the six-hour standard to those who did not (Stata, v.10.1; College Station, TX). Included in the logistic regression analysis were patient age and sex, presence of mental confusion (Y/N), chief complaint at triage and to doctor, triage vital signs, and co-morbidities. In addition, because previous studies have shown that ED crowding can affect antibiotic administration times [6, 7], we included day of week and time of day. Time of day was categorized as early (7 AM - 2:59 PM), midday (3 PM to 10:59 PM), and night (11 PM to 6:59 AM). Statistical significance was set at P < or = 0.05.

## Results

**Figure 1:**
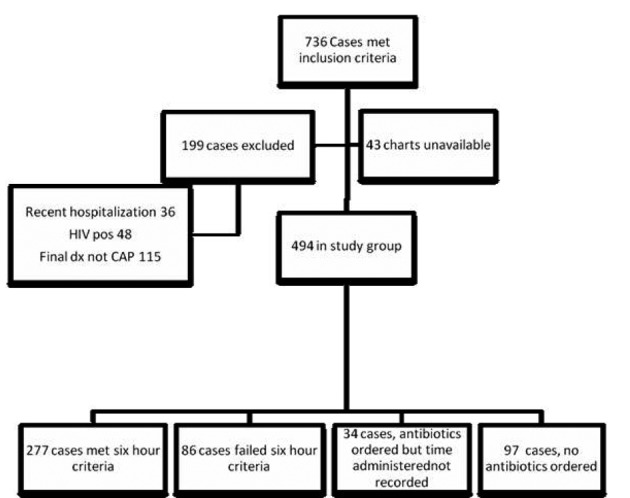
Included and excluded cases.

A total of 736 cases met the inclusion criteria; 199 cases met exclusion criteria; 43 charts were unavailable, leaving 494 cases as the study group. Among the included cases, 363 cases had complete antibiotic time records while 131 cases had incomplete records (Fig. 1).

From univariate analysis of patients with complete antibiotic data, the only characteristics meeting Bonferroni criteria for statistical significance were respiratory rate and average oxygen saturation (P = 0.002 and P < 0.001, respectively) (Table 1). Mean pulse rate approached statistical significance (P = 0.007) and there were no statistically significant differences for any other vital signs, co-morbidities, chief complaints, day-of-week or hour of day of presentation.

**Table 1:** Univariate Analysis Comparing Characteristics of Cases That Met and Failed to Meet the Joint Commission Six-Hour Standard for Antibiotic Administration

Variable	Met criteria (n = 277)	Failed to meet criteria (n = 86)	P value
Age (mean ± SD)	63.2 ± 18.7	60.9 ± 17.3	0.32
Female, %	49	52	0.62
Triage vital signs (mean ± SD)			
Pulse	111 ± 22	104 ± 17	0.007
Systolic BP	140 ± 31	141 ± 27	0.76
Respiratory rate	25 ± 9	22 ± 6	0.002
Temperature	99.9 ± 2.2	99.6 ± 1.9	0.22
Oxygen saturation	87 ± 10	92 ± 5	< 0.001
Nursing home resident, %	2.9	1.2	0.69
Altered mental status present, %	12.6	5.8	0.11
Co-morbidities, %			
History of liver disease	4.7	5.8	0.78
History of CHF	6.1	4.7	0.79
History of cardiovascular accident	9.8	9.3	1.0
History of renal disease	5.0	1.2	0.21
History of cancer	9.4	7.0	0.66
Chief complaint at triage, %			
Fever	22.0	24.4	0.66
Dyspnea	40.8	29.1	0.06
Cough	39.7	34.9	0.45
Chest pain	22.7	23.3	1.0
Weakness/fatigue	11.9	12.8	0.85
Abdominal pain	3.3	7.0	0.13
Chief complaint to doctor, %			
Fever	45.1	40.7	0.54
Dyspnea	48.7	43.0	0.39
Cough	61.0	64.0	0.70
Chest pain	31.8	32.6	0.90
Weakness/fatigue	11.6	10.5	0.85
Abdominal pain	7.6	7.0	1.0
Day of week, %			
Sunday	10.1	8.1	0.21
Monday	15.9	17.4	
Tuesday	14.4	23.3	
Wednesday	15.2	15.1	
Thursday	19.9	9.3	
Friday	13.4	12.8	
Saturday	11.2	14.0	
Time of day, %			
Early (7:00 - 14:59)	42.2	41.9	0.05
Mid-day (15:00 - 22:59)	36.8	47.7	
Night (23:00 - 6:59)	20.9	10.5	

Multiple logistic regression identified five variables associated with time to antibiotic administration: triage pulse rate, oxygen saturation, presence of altered mental status, hour of day, and day of week (Table 2). Chances for meeting the six-hour standard were increased if the person exhibited altered mental status, if their oxygen saturation was decreased, or if their pulse rate was elevated. If the person had altered mental status they were > 3.5 times more likely to meet the six-hour criteria. A 1% decrease in O^2^ saturation and 5 beat increases in pulse rate were each associated with a 10% increase in chances to meet criteria. Patients who presented to the ED between 3 PM and 11 PM were only one-third as likely to meet the JC criteria as those who presented early in the day or late at night. If they presented on a Thursday, they were 2.8 times more likely to meet the six-hour criteria.

**Table 2:** Results of Multiple Logistic Regression Using Stepwise Variable Selection to Identify Patient Risk Factors Associated With Likelihood to Meet Joint Commission Criteria for Administration of Antibiotics Within Six Hours if Diagnosed With CAP in the ED

Risk factors	Odds ratio	(95% CI)	P value
Pulse rate	1.02	1.00 - 1.03	0.010
Oxygen saturation	0.90	0.85 - 0.95	< 0.001
Altered mental status present	3.63	1.14 - 11.51	0.029
Presentation 3PM-11PM	0.35	0.14 - 0.89	0.027
Presentation on Thursday	2.83	1.16 - 6.91	0.023

Because 27% of the included cases had incomplete antibiotic data and so were not included in the logistic regression, we compared the characteristics of the complete-data versus incomplete-data groups to rule out the possibility that those with incomplete data might constitute a distinct group. From univariate analysis, the only characteristic meeting Bonferroni criteria for statistical significance was average oxygen saturation which was lower in the complete data group (88 ± 9% vs 91 ± 7%, P = 0.002) (Table 3). Average temperature and pulse rate approached statistic al significance but the differences were not clinically significant (Table 3). A larger percentage of the patients with complete antibiotic data reported a fever to the doctor (44% vs 31%, P = 0.007) (Table 3). Otherwise, the cases with incomplete antibiotic data appear to be similar to those included in the logistic regression analysis.

**Table 3:** Characteristics of Cases Included in Multiple Logistic Regression Compared to Cases not Included due to Incomplete Antibiotic Data

Variable	Cases with complete antibiotic data (n = 363)	Cases with incomplete antibiotic data (n = 131)	P value
Age (mean ± SD)	62.7 ± 18.4	61 ± 17.5	0.37
Female, %	50	47	0.68
Triage vital signs (mean ± SD)			
Pulse	109 ± 21	104 ± 20	0.008
Systolic BP	140 ± 30	143 ± 27	0.45
Respiratory rate	24 ± 8	23 ± 6	0.19
Temperature	99.8 ± 2.2	99.2 ± 1.9	0.006
Oxygen saturation	88 ± 9	91 ± 7	0.002
Nursing home resident, %	2.5	1.5	0.74
Altered mental status present, %	11.0	8.4	0.50
Co-morbidities, %			
History of liver disease	5.0	6.1	0.65
History of CHF	5.8	8.4	0.30
History of cardiovascular accident	9.6	8.4	0.73
History of renal disease	4.1	3.1	0.79
History of cancer	8.8	9.2	0.86
Chief complaint at triage, %			
Fever	22.6	13.0	0.21
Dyspnea	38.0	37.4	0.92
Cough	38.6	35.9	0.60
Chest pain	22.9	24.4	0.72
Weakness/fatigue	12.1	9.2	0.42
Abdominal pain	4.1	6.1	0.34
Chief complaint to doctor, %			
Fever	44.1	30.5	0.007
Dyspnea	47.4	42.0	0.31
Cough	61.7	51.9	0.06
Chest pain	32.0	35.1	0.52
Weakness/fatigue	11.3	10.7	1.0
Abdominal pain	7.4	7.6	1.0
Day of week, %			
Sunday	9.6	16.8	0.18
Monday	16.3	13.7	
Tuesday	16.5	12.2	
Wednesday	15.2	13.7	
Thursday	17.4	12.2	
Friday	13.2	15.3	
Saturday	11.9	16.0	
Time of day, %			
Early (7:00 - 14:59)	42.2	48.9	0.06
Mid-day (15:00 - 22:59)	39.4	41.2	
Night (23:00 - 6:59)	18.5	9.9	

## Discussion

Our results indicate that triage pulse rate, triage oxygen saturation level, presence of altered mental status, hour of day and day of week of presentation are each associated with the likelihood of receiving antibiotics within 6 hours of presentation for patients diagnosed with CAP in our ED. Patients who demonstrated altered mental status, lower oxygen saturation or an increased heart rate early in their patient encounter are more likely to receive antibiotics within six hours than those with vital signs closer to normal. Patients who present during the late afternoon and evening (3 - 11 PM) have reduced chances of meeting the six-hour criteria while those who present on Thursdays have increased chances.

It stands to reason that patients with elevated pulse rates, reduced oxygen saturation, and altered mental status are more likely to meet the six-hour standard because there is little doubt that they are sick and the decision to give antibiotics does not necessarily require laboratory or imaging tests. Our results are in agreement with previously published studies that have also identified associations between ‘markers of severe illness’ and increased odds of meeting antibiotic administration guidelines, including reduced oxygen saturation [7, 13, 14], elevated pulse rate [7, 14], elevated respiratory rate [7, 14], and fever [14]. There is disagreement however on the role of altered mental status. The results of the present study appear to group altered mental status with other signs of sepsis thereby contributing to reduced time to antibiotic administration. This conflicts with the findings of Waterer et al who found that altered mental status was associated with delays in antibiotic administration [5]. This incongruity is likely to be due to the somewhat different definitions of altered mental status in the two studies and the differences in inclusion criteria for the two studies (patients with clinical evidence of pneumonia vs patients with ED diagnosis of pneumonia).

In the absence of obvious signs of pneumonia such as fever and hypoxia, the time to antibiotic administration trends toward times in excess of four hours [5, 15]. When diagnostic uncertainty arises, such as when patients with CAP lack rales or hypoxia, laboratory and imaging studies must be completed before a definitive diagnosis can be made. Up to 22% of Medicare patients in one study presented in a manner likely to result in delayed diagnosis of CAP [15].

ED crowding has been shown to negatively impact the timely administration of antibiotics to CAP patients [6, 7]. In the present study, the association between time of day of presentation and time to antibiotic administration is likely due to ED crowding because peak ED patient census generally occurs between 3 PM and 11 PM in our facility. Significant factors influencing ED crowding are the number of people in the waiting room and the average length of stay for admitted paitents [7]. In our facility, the number of people in the waiting room increases steadily from about 10 AM and the number of available ED beds decreases as admitted patients wait to be moved to the floor. Although these previous studies examined compliance with the older 4-hr standard, we believe the general trends regarding the effects of ED crowding and timing of antibiotic administration are applicable to the current study. The association of Thursdays with reduced time to antibiotic administration is also likely to be due ED crowding as Thursdays are generally our lowest census days.

### Limitations

This study relied on retrospective chart review for data and consequently was limited solely to what was recorded. For this study, if the time for drug administration was not recorded, we assumed it was not given. In addition, it was conducted at a single institution which is a county hospital and the results of the study may or may not be generalizable to different hospital settings. Similarly, our patient population may be unusual in that few have third party insurance and a significant proportion has limited access to primary care. Consequently many of our patients present to the ED in advanced stages of illness and require hospital admission. This could increase the proportion of patients seen who have abnormal vital signs.

It is concerning that the final analysis of data for this study included just 45% of the cases that met inclusion criteria, but this is not out of line with previously published papers. In this realm of investigation, the inclusion criteria are often very broad and then the exclusion criteria are applied to pare down the number of cases that apply to the question of interest. It is not unusual to find that the study population remaining after the application of all exclusion criteria can range from 46 to 64% of the original number of included cases [1, 2, 6, 12, 16].

### Conclusions

Compared to patients who were given antibiotics within six hours of presentation, those who have prolonged times for antibiotic administration are likely to have pulse rates and oxygen saturation levels at presentation that are closer to normal, thus contributing to diagnostic uncertainty for CAP. They are also likely to present to the ED during the time of day when it is most crowded. The likelihood to meet JC criteria was improved if a patient’s oxygen saturation was below normal, if pulse rate was elevated, if mental confusion was evident, or if ED presentation was very early or very late the day, or on a lower census day.

## Declaration

Presented at the Western Regional SAEM meeting, February 2011, Keystone CO; Paul L Foster School of Medicine Research Colloquium, May 2011, El Paso TX; SAEM Annual Meeting, June 2011, Boston MA.
